# Alpha-1-Antitrypsin in Pathogenesis of Hepatocellular Carcinoma

**DOI:** 10.5812/hepatmon.7042

**Published:** 2012-10-30

**Authors:** Aleksandra Topic, Mila Ljujic, Dragica Radojkovic

**Affiliations:** 1 University of Belgrade, Faculty of Pharmacy, Department of Medical Biochemistry, Belgrade, Serbia; 2 University of Belgrade, Institute of Molecular Genetics and Genetic Engineering, Belgrade, Serbia

**Keywords:** Alpha 1-Antitrypsin Deficiency, Carcinoma, Hepatocellular, Autophagy, Fucose

## Abstract

**Context:**

Alpha-1-antitrypsin (A1AT) is the most abundant liver-derived, highly polymorphic, glycoprotein in plasma. Hereditary deficiency of alpha-1-antitrypsin in plasma (A1ATD) is a consequence of accumulation of polymers of A1AT mutants in endoplasmic reticulum of hepatocytes and other A1AT-producing cells. One of the clinical manifestations of A1ATD is liver disease in childhood and cirrhosis and/or hepatocellular carcinoma (HCC) in adulthood. Epidemiology and pathophysiology of liver failure in early childhood caused by A1ATD are well known, but the association with hepatocellular carcinoma is not clarified. The aim of this article is to review different aspects of association between A1AT variants and hepatocellular carcinoma, with emphasis on the epidemiology and molecular pathogenesis. The significance of A1AT as a biomarker in the diagnosis of HCC is also discussed.

**Evidence Acquisitions:**

Search for relevant articles were performed through Pub Med, HighWire, and Science Direct using the keywords “alpha-1-antitrypsin”, “liver diseases”, “hepatocellular carcinoma”, “SERPINA1”. Articles published until 2011 were reviewed.

**Results:**

Epidemiology studies revealed that severe A1ATD is a significant risk factor for cirrhosis and HCC unrelated to the presence of HBV or HCV infections. However, predisposition to HCC in moderate A1ATD is rare, and probably happens in combination with HBV and/or HCV infections or other unknown risk factors. It is assumed that accumulation of polymers of A1ATD variants in endoplasmic reticulum of hepatocytes leads to damage of hepatocytes by gain-of-function mechanism. Also, increased level of A1AT was recognized as diagnostic and prognostic marker of HCC.

**Conclusions:**

Clarification of a carcinogenic role for A1ATD and identification of proinflammatory or some still unknown factors that lead to increased susceptibility to HCC associated with A1ATD may contribute to a better understanding of hepatic carcinogenesis and to the development of new drugs.

## 1. Context

Alpha-1-antitrypsin (A1AT) is the most abundant liver-derived glycoprotein in plasma and predominant circulatory protease inhibitor. It is also an acute phase reactant and its plasma concentrations increase three to five-fold during the host response to inflammation/tissue injury. A1AT is the archetype member of the SERPIN (SERine Proteinase INhibitor) super family of structurally related proteins, which have remarkable structural homology characterized by a dominant A β-sheet and a mobile reactive center loop that presents a peptide sequence as the pseudosubstrate for the target proteinase (1). According to the serpin nomenclature it has been marked as SERPINA1 (serine proteinase inhibitor, clade A, member 1). The hepatocytes are the main source of A1AT, and the SERPINA1 gene is under control of different cytokines, such as interleukin-1 (IL-1), tumour necrosis factors α (TNFα), and the most effective interleukin-6 family of cytokines (interleukin-6, leukaemia inhibitory factor, oncostatin M) (2). The SERPINA1 gene is located on the protease inhibitor (Pi) locus, on the long arm of chromosome 14, mapped to 14q31–32, and shows a co-dominant pattern of inheritance (3). Following maturation, A1AT is secreted into the circulation as a 52-kD single-chain glycoprotein composed of 394 amino acid residues and 3 asparagine-linked complex carbohydrate side chains. The internal structure of A1AT is highly ordered, with nine α-helices (A→I) and three β-sheets. One of the most striking features of the serpins, including A1AT, is their dramatic conformational rearrangement during the inhibition of target proteinase. Upon binding of target proteinase, reactive center of the proteinase is cleaved and proteinase is transferred to other end of the serpin molecule. This conformational change is required for a serpin to function as a proteinase inhibitor, but can also be detrimental because point mutations facilitate the sequential insertion of the reactive site loop into a β-sheet of another molecule, thereby leading to formation of polymers.

The Pi locus is highly polymorphic and the most common alleles of A1AT are M1 (Ala213), M1 (Val213), M2 and M3, which have a normal serum level (1.5-3.5 g/L) and ability to inhibit target proteinases. Despite a great number of A1AT variants, only a few are clinically relevant, and assigned as deficient A1AT variants (A1ATD). Deficiency of A1AT is the most common genetic cause of early-onset panlobular emphysema, liver disease in childhood, and can also manifest with cirrhosis and/or hepatocellular carcinoma in adulthood. Some of the deficient variants accumulate in intracellular endoplasmic reticulum of hepatocytes and other A1AT-producing cells and therefore are inefficiently secreted. Several point mutations of A1AT are known to cause a perturbation in protein structure with consequent polymerization and intracellular accumulation. Mutations responsible for the molecular instability of the protein occur in the hinges and sliding regions involved in the movement of reactive loop to other end of molecule (4). The effect of these mutations allows spontaneous opening of the main β sheet of the molecule which results in rapid insertion into the sheet of the reactive loop of the next molecule and formation of loop-sheet polymers. Protein aggregation in the liver occur in patients homozygous for most common mutated variant - Z (5, 6) and two other rare variants - Siiyama (7) and Mmalton (8, 9). Histological hallmark of liver disease in A1AT deficiency is the presence of A1AT-containing globules positive to diastase-resistant periodic acid-Schiff (PAS-D) staining in some, but not all hepatocytes. Link between A1AT and liver malignancy has been investigated from several aspects, mainly by association of A1AT polymorphisms, elevation of serum levels, and changes in oligosaccharide content of A1AT with different liver disease, from cirrhosis to hepatocellular carcinoma (HCC).

## 2. Evidence Acquisition

Search for relevant articles were performed through Pub Med, HighWire, and Science Direct using the keywords “alpha-1-antitrypsin”, “liver diseases”, “hepatocellular carcinoma”, and “SERPINA1”. Articles published until 2011 were reviewed.

## 3. Results

### 3.1. Liver Disease Epidemiology and Polymorphism of A1AT in Adulthood

Epidemiological evidence and clinical features of liver disease in adult-carriers of A1ATD show marked variability in phenotypic expression of liver disease among affected PiZZ homozygotes. Relatively little is known about the factors that predispose subpopulation of PiZZ individuals to liver disease and/or protect remainder of the PiZZ population from liver disease (10, 11). Furthermore, cirrhosis in adult A1ATD Z-allele carriers can occur without preceding history of childhood liver disease (12). Variations in onset and severity of liver disease suggest that genetic and environmental modifiers have dramatic effects on clinical manifestations of liver disease. A prospective Swedish study showed that only approximately 10% of PiZZ and PiSZ 26-year-old subjects had marginal deviations in liver test results (13). Generally, A1ATD adults show clinical manifestations of chronic liver disease during middle or old age. Long-term observation (14) revealed slow progression of chronic liver disease in A1ATD adults. The mean ages of patients with advanced liver disease were 58, 66, and 73 years for PiZZ, PiSZ, and PiMZ phenotypes, respectively, and in 40% of these patients the survival was less than two years (15). Two Swedish autopsy studies conducted on PiZZ patients revealed that over one third of elderly patients have developed cirrhosis and primary liver cancer (16, 17).

Several studies have shown that PiMZ in heterozygous state may lead to chronic liver disease, cryptogenic cirrhosis, and chronic active hepatitis (18-20). However, in a large case-control study (21) no association was found between A1ATD heterozygotes and chronic liver disease, while higher prevalence of PiMZ in decompensated liver disease due to presence of HCV infection or non-alcoholic fatty liver disease. Propst et al. (22) also observed the co-morbidity of viral infection and A1ATD with chronic liver disease in a cross-sectional study. Moreover, Eigenbrodt et al. (23) provided evidence of an association between Z heterozygosity and end stage liver diseases of several etiologies such as HCV infection, alcoholic liver disease, primary hepatic malignancy, and cryptogenic cirrhosis. In contrast, Bowlus et al. (24) found that male gender and obesity, but not alcohol or viral hepatitis, predispose advanced liver disease in adults with A1ATD. A recent study reported that S and Z A1AT variants are associated with hyperferritinemia and sinusoidal iron accumulation, but not with severe liver damage in non-alcoholic fatty liver disease (25).

Case reports and family studies also reported association between liver disease and some of rare A1AT variants such as Mduarte (26), S allele heterozygosity (27, 28), and M3 allele (29). In addition to cirrhosis, hepatocellular carcinoma is one of the possible complications that could occur in A1ATD adults with chronic liver disease. It is suggested that risk for HCC in A1ATD individuals could not be fully attributed to cirrhosis, but rather could represent a downstream effect of A1AT intracellular retention and accumulation (30). Generally, A1ATD variants could be associated with HCC in two ways: as an independent genetic risk factor for HCC, or as a risk factor for hepatoviral infections that cause advanced liver disease and consequently hepatocellular carcinoma.

First data of association between A1ATD and HCC were obtained in the seventies of 20th century (31). Two Scandinavian studies revealed that PiZZ patients with cirrhosis over 50 years of age have a significant risk to develop HCC (16, 32). Specific A1AT immunoreactivity was identified in tumor cells, thus confirming an association between hepatic deposition of A1AT and occurrence of primary liver carcinoma (33-35). Unique and important epidemiologic population-based retrospective study which collected data from all autopsies performed during 20-years in Malmö, Sweden demonstrated an overall increased risk of cirrhosis and primary liver cancer in PiZZ males (36). A case-control study conducted also in Malmö, Sweden, confirmed that PiZZ males are at greater risk of cirrhosis and HCC unrelated to the presence of HBV or HCV infections (37). Predisposition to HCC in PiMZ heterozygotes is rare, and probably happens in combination with HBV and/or HCV infections or other unknown risk factors. Propst et al. (38) showed that A1ATD heterozygotes with cirrhosis have as high risk of developing HCC as those with other causes of cirrhosis. Their research has shown high prevalence of viral infection in A1ATD-associated chronic liver disease leading to cirrhosis and furthermore to the development of HCC. Therefore, they recommended vaccination against HBV and when available against HCV infections. However, recent studies have shown that Z heterozygosity-associated HCC may develop in non-cirrhotic liver tissue, and frequently as cholangiocarcinoma (39, 40). In contrast, studies in different populations showed that association between A1ATD phenotypes and HCC is either non-existent or very weak (41-45). In a review article, Sherman (46) was concluded that there was insufficient data to determine whether screening for HCC might be beneficial for patients with cirrhosis caused by A1ATD. Several case-report studies have reported associations between A1ATD and HCC: a 16-year-old PiMZ boy with hepatocellular carcinoma (47); a PiMZ patient with hepatocellular carcinoma in a non-cirrhotic liver (48); and a primary liver cell carcinoma as a complication in a 67-year-old PiZZ patient (49). However, in the case of PiMZ patient with cirrhosis and hepatocellular carcinoma, histology revealed A1AT globules in hepatocytes located at the periphery of the cirrhotic nodules, while they were sparse within neoplastic hepatocytes (50).

Association between non-A1ATD variants and HCC was also found in two studies which showed high prevalence of rare PiMF variant (41, 51).

### 3.2. Molecular Mechanism of Liver Injury in A1ATD

Accumulation of polymers of A1ATD variants in endoplasmic reticulum (ER) of hepatocytes leads to damage of hepatocytes by gain-of-function mechanism accompanied by plasma protein deficiency. Confirmation on gain-of-toxic function as the mechanism by which accumulation of protein damages the cell comes from the observation that in mice transgenic for Z mutant of human A1AT (A1AT-Z) gene hepatic inflammation and carcinoma developed (52). As these mice had normal levels of endogenous anti-proteinases, the liver disease was attributable to a gain-of-toxic function mechanism. Transgenic young A1AT-Z mice expressing higher serum levels of A1AT were more likely to develop tumors with age than those with lower levels of A1AT (53). This study also showed that accumulation of A1AT-Z altered regulation of several genes, including cyclin D1 and MCAM, which lead to cell proliferation and tumorigenesis.

Study of genetically engineered skin fibroblast cell lines from PiZZ individuals with or without liver disease indicated that there was a lag in ER degradation of A1AT-Z (54). Moreover inefficient degradation of A1AT-Z in the ER was in correlation with susceptibility to liver disease. These results suggested that modifiers of the disease affect the pathways responsible for disposal of A1AT-Z and thereby attention of researchers was directed toward these pathways. Two explanations for the effects of such modifiers have been postulated: variation in the function of intracellular degradation mechanisms and/or variation in signal transduction pathways activated to protect the cell from protein mislocalization and/or aggregation (55). Expression of mutant proteins disrupts protein folding in the ER and causes cellular response known as ER stress. Terminally misfolded proteins are selectively transported from the ER into the cytosol, and are subsequently ubiquinated and degraded by proteasome in a process called ER-associated degradation (ERAD). In the absence of efficient protein degradation, or if accumulation of misfolded proteins in the ER overwhelms degradation machinery, several ER response pathways can be activated. Unfolded protein response (UPR) is a signal transduction pathway that activates a wide spectrum of genes in response to accumulation of unfolded, misfolded, or unassembled proteins in the ER and decrease translation initiation in such a way that only specific mRNAs can be translated.

Signs of the unfolded protein response were found neither in A1AT-Z stable transfected cell line, nor in transgenic mice (10). Using HeLa and murine hepatoma cell lines as a model and investigating synthesis of BiP and CHOP (UPR targets) showed that accumulation of A1AT-Z in the ER did not activate UPR. Expression of A1ATSaar variant (carboxyl terminal tail is truncated) which is retained in ER but does not form polymers was found to induce UPR, suggesting that the lack of activation of UPR by ER retention of A1AT-Z protein somehow influences propensity of A1AT-Z to form insoluble polymers in ER. On the other hand, work from Carroll et al., indicates that UPR is activated by A1AT-Z in human peripheral blood monocytes, suggesting that species- and cell-specific differences may exist (56). 

There are at least two pathways for degradation of Z variant that accumulates in ER: proteasomal degradation pathway and autophagy. While proteasome is responsible for degrading soluble forms of Z variant (57), autophagy is specialized for disposal of insoluble polymers and aggregates. Experiments with yeast A1AT-Z expression system discovered that, at low levels of expression, A1AT-Z remained mainly soluble and could be handled by ERAD pathway. In contrast, higher levels of A1AT-Z resulted in formation of polymers and a functional autophagic pathway was required for degradation and cell viability (58). As a result, triggering autophagy induction by A1AT-Z might be caused by formation of polymers. Results of two studies by Teckmann et al. (11, 59) indicate that retention of A1AT-Z in ER is associated with a marked autophagic response. Using genetically engineered A1AT-Z mouse as an in vivo model, and fasting as an environmental stressor, they showed that autophagy is constitutively activated and that fasting does not lead to an increase in autophagosomes in the liver of PiZ mouse. A hypothesis proposed by the authors implicating that autophagic response exhibits a protective role by clearing ER with aggregated mutant A1AT-Z molecules and that protective mechanism of A1AT-Z liver is very weak and would probably be easily overwhelmed by physiological and pathological stressors.

**Figure 1:**
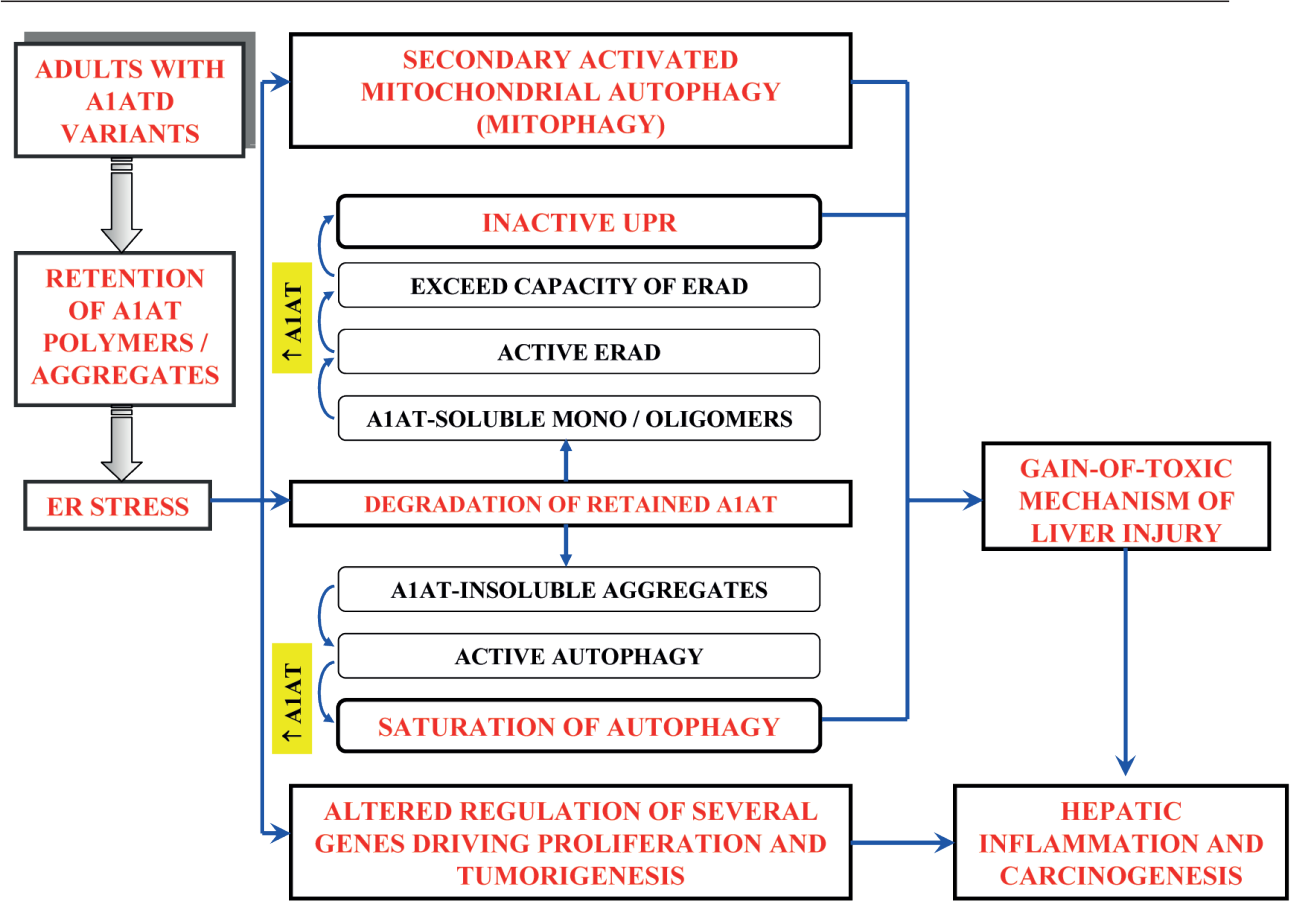
Possible Mechanisms Responsible for Hepatocellular Carcinoma in A1ATD A1ATD = alpha-1-antitrypsin deficiency; ER = endoplasmic reticulum; ERAD = ER-associated degradation; UPR = unfolded protein response

Mitochondrial autophagy and mitochondrial injury are also present in the liver in A1AT deficiency, which provides evidence that mitochondrial dysfunction is involved in liver cell injury mechanism in A1AT deficiency (60, 61). Since autophagic response participates in both degradation of A1AT-Z and in cellular response to accumulation of A1AT-Z in ER, drugs enhancing autophagy, such as carbamazepine, could be potentially used to prevent hapatotoxicity due to A1AT deficiency (55). Accumulation of aggregation prone to A1AT variants in ER may potentially induce multiple signaling events related to ER stress. Given the heterogeneity of liver disease in A1AT deficiency, it can be hypothesized that clinically significant liver damage occurs only in A1AT-deficient patients who also have some other defect in ER quality control and that these defects are heterogeneous among the affected population. Considering previously mentioned studies, the possible mechanisms responsible for hepatocellular carcinoma in A1ATD are shown in Figure 1.

Whereas relatively little is known about the pathogenesis of hepatocellular carcinoma associated with A1ATD, future research will provide answers to many unresolved issues. A detailed elucidation of the mechanisms by which aggregated A1AT Z is degraded in ER is essential to understand how the quality control of ER works in general, and to recognize the specific issue of how a subgroup of A1AT deficient individuals becomes susceptible to liver injury and carcinogenesis.

### 3.3. A1AT as Biomarker for Hepatocellular Carcinoma

Elevated level of A1AT is suggested as cancer marker that discriminates cancer from chronic benign diseases, and clinical remission from relapse (62). A recent study (63) using proteomics identified that A1AT, along with 50 other proteins, showed marked difference between the HCC tissue samples and pre-cancerous lesions, suggesting that alterations in protein expression occurred frequently during the process of hepatocarcinogenesis. Increased level of A1AT was recognized as a diagnostic and prognostic marker of HCC (64, 65). Serum A1AT level in patients with HCC was significantly higher than those in patients with liver cirrhosis or chronic hepatitis (66). The exact mechanism and role of elevated serum level of A1AT in HCC is still unclear. Results of the study by Sawaya et al. (67) supported a hypotheses that production of A1AT by tumor cells correlates with the regional proteolytic and inflammatory activity, which are probably involved in the protection of tumor cells.

In addition, a few studies indicated that serum levels of A1AT represent an important survival prognostic factor among patients with HCC. Increased levels of serum A1AT in HCC patients are in correlation with shorter survival, and a difference of serum A1AT of 0.02 g/L correlates with 25% shorter expected survival time , while a difference of 0.04 g/L implies more than 40% decrease in survival time (68). Similar results showed that HCC patients with A1AT levels > 2.20 g/L had significantly decreased median survival than patients with A1AT levels ≤ 2.20 g/L (69), suggesting that A1AT level is an independent predictor of survival.

In effort to found more sensitive and specific tumor marker for hepatocellular carcinoma, the levels of A1AT and levels of alpha-fetoprotein (AFP) were combined. These combined levels were found to be more sensitive and more specific tumor marker for hepatocellular carcinoma, than separate A1AT and AFP levels. The sensitivity to HCC using only AFP (> 400 ng/mL) or only A1AT level (> 3.2 g/L) were 52% and 76%, respectively while combined test sensitivity was improved to 80% (70). Changes in glycosylation of proteins, most notably fucosylation, have been associated with the development of hepatocellular carcinoma (71). It has been reported that elevated concentration of fucose acid was in strong positive correlation with serum level of A1AT, and was a measure of tumor spread (72, 73). Furthermore, human hepatoma cell line PLC/PRF/5 synthesized and secreted a functional A1AT with normal molecular size but with atypical, highly branched and incompletely sialylated carbohydrate chains (74).

Wang et al. (75) in searching for a reliable biomarker for early detection of HCC found that the level of fucosylated A1AT in combination with fucosylated kininogen, level of α-fetoprotein, and Golgi protein 73 (GP73) gives sensitivity of 95% and specificity of 70% in diagnosis HCC. A very recent study (76) reported in details the stepwise changes in glycosylation of A1AT progressing from liver cirrhosis to cancer and identified a core fucosylation on A1AT as a HCC specific modification.

## 4. Conclusions

To date, research on the role of alpha-1-antitrypsin in the ethiology and pathogenesis of hepatocellular cancer could be summarized in the following facts. This protein is useful diagnostic and prognostic marker of HCC, in qualitative (fucosylated A1AT) and quantitative sense (increased serum levels). On the other hand, epidemiological studies have shown an association between A1ATD and HCC. At first sight, the facts that both high levels and deficiency of the A1ATD are associated with HCC could be contradictory. However, A1ATD variants are normally synthesized, but they are trapped in endoplasmic reticulum of hepatocytes leading to hepatocyte damage by gain-of-function mechanism followed by plasma deficiency of the protein. In contrast, increased levels of serum A1AT in HCC patients are in correlation with shorter survival. The possible explanation is that elevated production of A1AT by tumor cells is involved in their protection.

Future investigations should clarify a role for A1ATD in carcinogenesis and identify pro-inflammatory or some still unknown factors that lead to increased susceptibility to HCC associated with A1ATD. Identifying these mechanisms will contribute to a better understanding of hepatic carcinogenesis, as well as to develop possible preventive measures.
